# *NKX2-5* variants screening in patients with atrial septal defect in Indonesia

**DOI:** 10.1186/s12920-022-01242-8

**Published:** 2022-04-22

**Authors:** Royhan Rozqie, Muhammad Gahan Satwiko, Dyah Wulan Anggrahini, Ahmad Hamim Sadewa, Gunadi, Anggoro Budi Hartopo, Hasanah Mumpuni, Lucia Kris Dinarti

**Affiliations:** 1 Department of Cardiology and Vascular Medicine, Faculty of Medicine, Public Health and Nursing, Universitas Gadjah Mada/Dr. Sardjito Hospital, Yogyakarta, 55281 Indonesia; 2 UGM Academic Hospital, Yogyakarta, 55291 Indonesia; 3 Department of Biochemistry, Faculty of Medicine, Public Health and Nursing, Universitas Gadjah Mada, Yogyakarta, Indonesia; 4 Pediatric Surgery Division, Department of Surgery/Genetics Working Group, Faculty of Medicine, Public Health and Nursing, Universitas Gadjah Mada/Dr. Sardjito Hospital, Yogyakarta, 55281 Indonesia

**Keywords:** *NKX2-5*, Familial ASD, Variant, Heterozygous variant, Arrhythmia, Pulmonary hypertension

## Abstract

**Background:**

*NKX2-5* variant in atrial septal defect patients has been reported. However, it is not yet been described in the Southeast Asian population. Here, we screened the *NKX2-5* variants in patients with atrial septal defect (ASD) in the Indonesian population.

**Method:**

We recruited 97 patients with ASD for genetic screening of the NKX2-5 variant using Sanger sequencing.

**Results:**

We identified three variants of *NKX2-5*: NM_004387.4:c.63A>G at exon 1, NM_004387.4:c.413G>A, and NM_004387.4:c.561G>C at exon 2. The first variant is commonly found (85.6%) and benign. The last two variants are heterozygous at the same locus. These variants are rare (3.1%) and novel. Interestingly, these variants were discovered in familial atrial septal defects with a spectrum of arrhythmia and severe pulmonary hypertension.

**Conclusion:**

Our study is the first report of the *NKX2-5* variant in ASD patients in the Southeast Asian population, including a novel heterozygous variant: NM_004387.4:c.413G>A and NM_004387.4:c.561G>C. These variants might contribute to familial ASD risk with arrhythmia and severe pulmonary hypertension. Functional studies are necessary to prove our findings.

**Supplementary Information:**

The online version contains supplementary material available at 10.1186/s12920-022-01242-8.

## Introduction

Congenital heart disease diagnosis and treatment capabilities have dramatically increased over the previous years, but equivalent success rates have yet to be established in developing countries like Indonesia. Since congenital heart disease accounts for almost a third of all congenital birth defects [1], focusing on it is critical to preventing unnecessary causes of death, such as pulmonary hypertension. However, most patients with congenital heart disease in developing countries lately seek medical attention as symptoms and signs of complications begin to manifest.

According to Indonesia’s first hospital-based registry of individuals with congenital heart disorders (CHD) and CHD-related pulmonary hypertension, atrial septal defects (ASD) account for most congenital heart diseases [2]. For decades, these patients may be asymptomatic. Although these patients may be identified coincidentally during routine checkups, there is no specific screening of asymptomatic CHD in Indonesia. Later symptoms will prompt individuals to contact a doctor and seek treatment at a hospital. Unfortunately, the patients’ primary complaints were associated with the development of pulmonary artery hypertension and resulted in a poor clinical outcome.

Genetic testing plays a significant role in medical evaluation. It detects possible genetic abnormalities in CHD and can enhance prognosis by providing vital information on individualized medical treatment and clinical diagnostic assurance [3]. Since 1950, genetic investigations on family congenital heart disease have been studied. Various studies since then have shown that genetic information has a role in structural heart abnormalities. Numerous genes, including *NKX2-5, GATA4, TBX5, NOTCH1*, and *TBX20,* have been related to congenital heart disease [4].

The transcription factor NKX2-5 plays an essential role in embryonic cardiogenesis and postnatal cardiac adaptation [5]. Previous studies have reported a genetic variant of *NKX2-5* linked to the development of atrial septal defect [6, 7]. The frequency of gene variant of NKX2-5 in ASD patients with familial history could reach up to 8% and only 1–4% in sporadic cases [8]. Familial genetic variants can be detected earlier, so the management is possible to be carried out before complications appear. In addition, gene variants are different among races. Therefore, more study is needed to identify the gene variant of *NKX2-5* in several different ethnicities. Here, we screened the NKX2-5 variants in patients with atrial septal defect in Indonesia.

## Material and methods

### Patient samples

This study was an analytic observational study involving ASD patients examined at RSUP Dr. Sardjito Yogyakarta and included in the COHARD-PH Registry in Yogyakarta [2]. It was sampled using the convenience sampling method. Informed consent was obtained from patients for diagnostic tests and research studies. The ethics committee approved the study of Universitas Gadjah Mada’s Faculty of Medicine, Public Health, and Nursing under the reference number KE/1116/11/2020. Storage and processing of DNA extraction from blood samples and the polymerase chain reaction were carried out at the Integrated Research Laboratory of the Faculty of Medicine, Public Health and Nursing Universitas Gadjah Mada.

### DNA extraction and Sanger sequencing

Genomic DNA was isolated from whole blood using a DNA extraction kit (Geneaid). DNA was extracted and kept at − 20 °C until it was analyzed. Polymerase chain reaction (PCR) was performed using a Promega PCR kit. The primers used in this study are described in Additional file 1.

Subsequently, the Sanger sequencing was performed for the screening of *NKX2-5* variants. The variants of *NKX2-5* were obtained after being compared with the sequences in the NCBI database using SerialCloner and Sequence Scanner software.

## Results

### Baseline characteristics

We included 97 ASD patients in this study. Subjects consisted of 25 familial ASD patients and 72 sporadic ASD patients. The participants in this study ranged from 13 to 79 years old, with an average of 36.04 years. The mean age in the familial and sporadic groups was 37.64 (± 13.06) years and 35.49 (± 13.88) years, respectively. There were more female patients than males in both groups, 21 patients (84.0%) in familial and 62 patients (86.1%) sporadic (Table 1).

**Table 1:** Sinus venosus is part of ASD type as secundum and primum

Characteristics	Familial (mean ± SD; n, %)	Sporadic (mean ± SD; n, %)	*p*-value
Age (years)	37.64 ± 13.06	35.49 ± 13.88	0.433
Sex			0.751
Male	4 (16)	10 (13.9)	
Female	21 (84)	62 (86.1)	
ASD type			0.621
Secundum	24 (96)	67 (93.1)	
Primum	0	3 (4.2)	
Sinus venosus	1 (4)	2 (2.8)	
Avg. defect diameter (mm)	26.3 ± 9.4	23.2 ± 7.12	0.141
Right atrium diameter (mm)	46.2 ± 5.82	46 ± 8.05	0.963
Mean pulmonary artery pressure (mmHg)	35.13 ± 17.27	40.39 ± 20	0.351
Eisenmenger syndrome	1 (4)	6 (8.3)	0.673
Arrhythmia	3 (12)	8 (11.1)	1.0

This study includes three types of defects: secundum defects, primum defects, and sinus venosus defects. The type of secundum defect was the most common in each group, 24 patients (96%) in the familial group and 67 patients (93.1%) in the sporadic group. Primum defect type was only found in the sporadic group, three patients (4.2%). Patients with sinus venosus defect type found one patient (4%) in the familial group and two (2.8%) in the non-familial group. The diameter of the ASD defect in the familial group was 26.3 (± 9.40) mm, while the diameter in the sporadic group was 23.2 (± 7.12) mm. Right atrial dilatation was 46.20 (± 5.72) mm in the familial group and 46.00 (± 8.05) mm in the sporadic group. Patients with ASD with severe pulmonary hypertension may develop Eisenmenger syndrome. Eisenmenger syndrome was diagnosed in one patient (4%) in the familial group and six in the non-familial group (8.3%) (Table 1).

### NKX2-5 variants in ASD patients

**Fig. 1:**
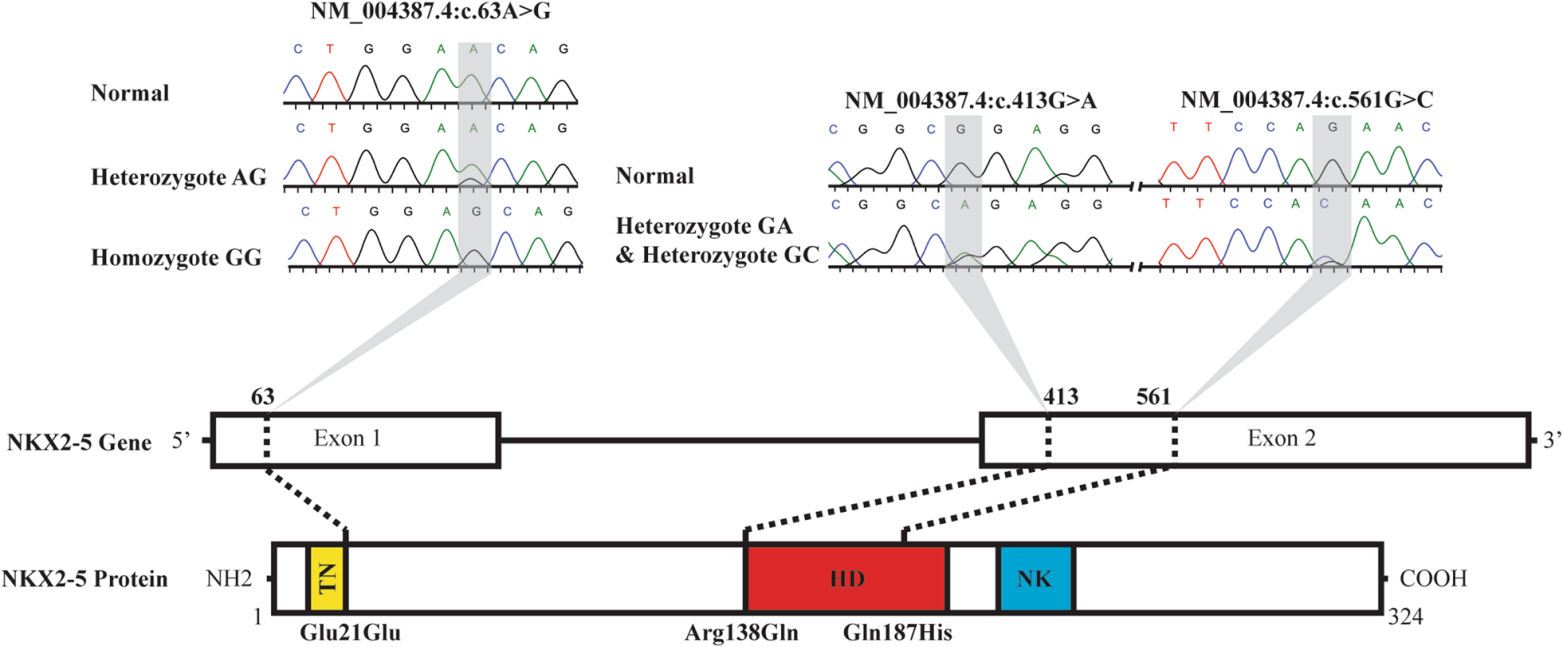
The results of NKX2-5 sequencing show three variants at the codons 63, 413, and 561. The first variant is NM_004387.4:c.63A>G at exon 1, consisting of heterozygous AG and homozygous GG. The second variant is a heterozygous GA (NM_004387.4:c.413G>A), and the last variant is a heterozygous GC (NM_004387.4:c.561G>C). The first variant is a synonymous variant. However, variants at codons 413 and 561 are non-synonymous (arginine replaced with glutamine at 138 and glutamine substituted with histidine at 187). Glu (glutamic acid), Arg (arginine), Gln (glutamine), His (histidine), NH2 (amino-end), TN (transcriptional activation domain), HD (homeodomain), NK (NK2 specific domain), COOH (carboxyl end)

During this study, 97 patients were subjected to *NKX2-5* screening using Sanger sequencing. From this study, three different variants were identified, which are NM_004387.4:c.63A>G at exon 1, NM_004387.4:c.413G>A, and NM_004387.4:c.561G>C at exon 2 (Fig. 1).

In our population study, the c.63A>G variant consists of 3 kinds of genotypes: homozygous AA (14, 14.4%), heterozygous AG (41, 42.3%), and homozygous GG (42, 43.3%) (Table 2). This variant was frequently observed in patients with ASD. According to ExAC, variant frequency is 40.52% in the control population [9].

**Table 2:** *NKX2-5* variants in our ASD patients

Variant	Genotype	Frequency (n, %)
NM_004387.4:c.63A>G	AA	14 (14.4)
	AG	41 (42.3)
	GG	42 (43.3)
NM_004387.4:c.413G>A	GG	94 (96.9)
	GA	3 (3.1)
	AA	–
NM_004387.4:c.561G>C	GG	94 (96.9)
	GC	3 (3.1)
	CC	–

**Fig. 2:**
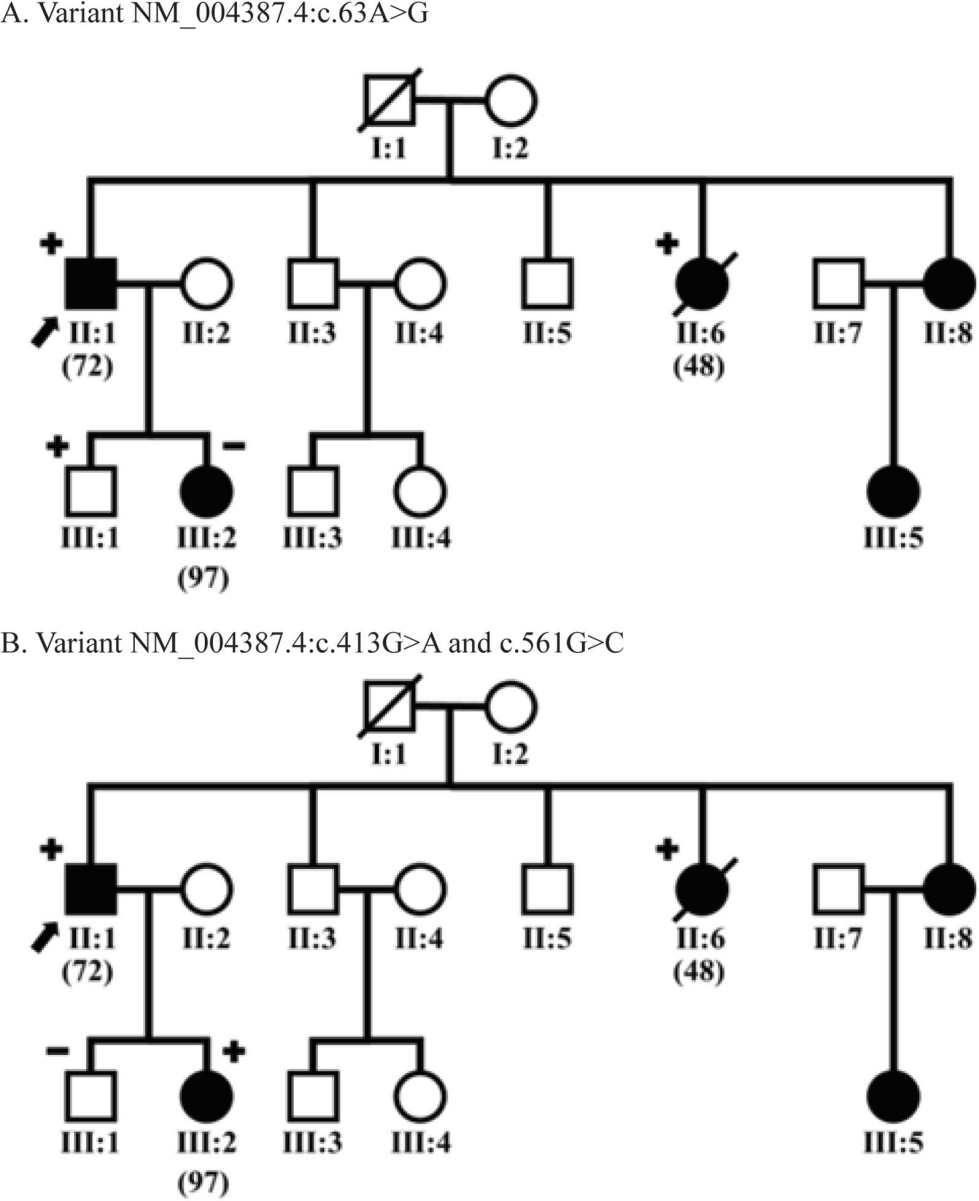
Patient’s family tree no. 48, 72, and 97. **A** describes the findings of the NM_004387.4:c.63A>G variant, while **B** depicts the dual variants of NM_004387.4:c.413G>A and NM_004387.4:c.561G>C. The square symbol denotes male, and the circle denotes female. Patient no. 72, in the index, is marked with an arrow. Black shading describes ASD patients. The + (positive) sign indicates a mutation, the − (negative) sign is no mutation. Subjects without a + or − sign indicate that genetics was not tested. The / (slash) sign indicates that the patient has died

The c.413G>A variant has two types of genotypes, homozygous GG and heterozygous GA. The c.561G>C variant also has two genotypes: homozygous GG and heterozygous GC. Notably, both heterozygous variants have been identified at the same locus. These double heterozygous variants may significantly impact the amino acid missense arrangement, which is very likely to change the structure and function of the NKX2-5 protein. These variants are remarkable because they occurred in three subjects (3.1%) of ASD patients from 1 family (Fig. 2). In the control population, the frequency of c.413G>A is 0.0009% in the GnomAD_exome database, and there is no data for c.561G>C [9].

## Discussion

Our study found several *NKX2-5* variants in ASD patients in Indonesia: NM_004387.4:c.63A>G at exon 1, NM_004387.4:c.413G>A, and NM_004387.4:c.561G>C at exon 2. The variant of c.63A>G is considered benign according to the ClinVar database. However, it is associated with a 20% decrease in transactivation activity [10]. In this study, it was frequently found that as much as 85.6% of research subjects. This result is the most significant number after research in the population in China, 80.18%, but it was found lower in Germany (59.4%) and Morocco (56.2%) [7, 8, 11, 12].

For c.413G>A variant, replacement of guanine to adenine is a non-synonym in the form of missense which changes the arginine (Arg) to glutamine (Gln). Whereas, for c.561G>C variant, guanine substitution to cytosine changes the amino acid glutamine (Gln) to histidine (His). These variants change the amino acid sequence and consequently modify the protein structure, which can interfere with the function of the NKX2-5 protein as a transcription factor.

The variants of c.413G>A and c.561G>C lay in amino acids at 138 and 187, respectively, part of the protein homeodomain. Variants change the amino acid arrangement in the homeodomain, which is a critical domain because it binds directly to specific DNA. Several previous studies reported that missense mutations in the homeodomain could cause secundum type ASD and familial conduction disorders [13–17].

In this study, we found these last two variants to occur together. It was found in 3 subjects with a family relationship (Fig. 2 and Additional file 1). Subjects 48 and 72 have sibling relationships, while subjects 72 and 97 are father and daughter. In subjects 48 and 72, it turned out that they had not only double heterozygote variants of c.413G>A and c.561G>C but also had c.63A>G heterozygous variant. It may be related to the more severe phenotype in subject 48, which developed rapid pulmonary hypertension aggravation and unfortunately led to her death. In addition, these double variants cause a phenotype in the form of ASD accompanied by arrhythmia disorders. Subject 72 had sinus node dysfunction at a young age, while subject 97 had atrial tachycardia during childhood and ablated. The previous study support that mutations in NKX2-5 cause atrial septal formation and arrhythmia disorders [18]. Therefore, it is necessary to do early genetic screening in the ASD patient family to see the role of the genetic variant of NKX2-5 on the familial ASD phenotype, especially in Indonesia.

Our study did not perform a functional analysis to determine the pathogenicity of the novel variant on ASD development. Therefore, further functional studies are necessary.

## Conclusion

Our study is the first report of the NKX2-5 variants in ASD patients in the Southeast Asian population, including novel heterozygous variants: NM_004387.4:c.413G>A and NM_004387.4:c.561G>C. These variants might contribute to familial ASD risk with arrhythmia and severe pulmonary hypertension. Functional studies are necessary to prove our findings.

## Supplementary Information

**Additional file 1**.** Table S1**. Primer Ex1-FW and Ex-RV are located in exon 1, while Ex2A-FW, Ex2A-RV, Ex2B-FW, and Ex2B-RV are located in exon 2.** Table S2**. AA – amino acid change; AT – atrial tachycardia; AVND – atrioventricular node dysfunction; Seq var – sequence variant; SND – sinus node dysfunction; SVT – supraventricular tachycardia.

## Data Availability

The genomic DNA sequences generated and/or analyzed during the current study are available in the GenBank repository, under the accession number OM066666-OM066670 and GitHub page (https://github.com/krisdinarti/NKX2-5_ASD_Indo).

## Abbreviation

ASD: Atrial septal defect
