# Supplementary material for: NKX2-5 variants screening in patients with atrial septal defect in Indonesia
Source: BMC Med Genomics. 2022 Apr 22;15:91. doi: 10.1186/s12920-022-01242-8 (PMC9027821; doi:10.1186/s12920-022-01242-8)
Supplement: Supplementary file 1 — Additional file 1. Table S1. Primer Ex1-FW and Ex-RV are located in exon 1, while Ex2A-FW, Ex2A-RV, Ex2B-FW, and Ex2B-RV are located in exon 2. Table S2. AA – amino acid change; AT – atrial tachycardia; AVND – atrioventricular node dysfunction; Seq var – sequence variant; SND – sinus node dysfunction; SVT – supraventricular tachycardia. [file 12920_2022_1242_MOESM1_ESM.docx]

**Supplementary**

Table S1. Primer for PCR

| Region | Primer name | Sequence | Length | Annealing temp |
| --- | --- | --- | --- | --- |
| 1 | Ex1-FW | 5'-CGGCACCATGCAGGGAAG-3' | 404 bp | 62℃ |
|  | Ex1-RV | 5'-AGGGTCCTTGGCTGGGTCGG-3' |  |  |
| 2A | Ex2A-FW | 5'-TGCAGAAGGCGGTGGAGC-3' | 336 bp | 63℃ |
|  | Ex2A-RV | 5'-CACTGGCACCGCGATCCT-3' |  |  |
| 2B | Ex2B-FW | 5ꞌ-AGAACCGGCGCTACAAGTG-3ꞌ | 473 bp | 60℃ |
|  | Ex2B-RV | 5ꞌ-GAGTCAGGGAGCTGTTGAGG-3ꞌ |  |  |

Primer Ex1-FW and Ex-RV are located in exon 1, while Ex2A-FW, Ex2A-RV, Ex2B-FW, and Ex2B-RV are located in exon 2.

Table S2. Summary of patient profile

| **Patient** | **Age** |  |  | **NKX2.5 exon 1** | | | **NKX2.5 exon 2A** | | | **NKX2.5 exon 2B** | | | |
| --- | --- | --- | --- | --- | --- | --- | --- | --- | --- | --- | --- | --- | --- |
| **No** | **(yrs)** | **Type of ASD** | **Arrhythmia** | **AA** | **Seq var** | **Zygos** | **AA** | **Seq var** | **Zygos** | **AA** | **Seq var** | **Zygos** |  |
| 1 | 32 | Secundum | None | Glu21Glu | A63G | Hetero | None | None |  | None | None |  |  |
| 2 | 26 | Secundum | None | Glu21Glu | A63G | Homo | None | None |  | None | None |  |  |
| 3 | 21 | Secundum | None | Glu21Glu | A63G | Hetero | None | None |  | None | None |  |  |
| 4 | 57 | Secundum | None | Glu21Glu | A63G | Homo | None | None |  | None | None |  |  |
| 5 | 30 | Secundum | None | Glu21Glu | A63G | Homo | None | None |  | None | None |  |  |
| 6 | 22 | Secundum | None | Glu21Glu | A63G | Homo | None | None |  | None | None |  |  |
| 7 | 22 | Secundum | None | Glu21Glu | A63G | Hetero | None | None |  | None | None |  |  |
| 8 | 30 | Secundum | None | None | None |  | None | None |  | None | None |  |  |
| 9 | 79 | Secundum | None | None | None |  | None | None |  | None | None |  |  |
| 10 | 53 | Secundum | Atrial fibrilation | Glu21Glu | A63G | Homo | None | None |  | None | None |  |  |
| 11 | 49 | Secundum | None | None | None |  | None | None |  | None | None |  |  |
| 12 | 49 | Secundum | None | Glu21Glu | A63G | Hetero | None | None |  | None | None |  |  |
| 13 | 66 | Secundum | Atrial fibrilation | Glu21Glu | A63G | Homo | None | None |  | None | None |  |  |
| 14 | 21 | Secundum | None | Glu21Glu | A63G | Hetero | None | None |  | None | None |  |  |
| 15 | 28 | Secundum | None | None | None |  | None | None |  | None | None |  |  |
| 16 | 30 | Secundum | None | Glu21Glu | A63G | Hetero | None | None |  | None | None |  |  |
| 17 | 55 | Secundum | None | Glu21Glu | A63G | Hetero | None | None |  | None | None |  |  |
| 18 | 28 | Secundum | None | Glu21Glu | A63G | Hetero | None | None |  | None | None |  |  |
| 19 | 51 | Secundum | None | Glu21Glu | A63G | Hetero | None | None |  | None | None |  |  |
| 20 | 50 | Secundum | None | Glu21Glu | A63G | Hetero | None | None |  | None | None |  |  |
| 21 | 35 | Secundum | None | Glu21Glu | A63G | Hetero | None | None |  | None | None |  |  |
| 22 | 44 | Secundum | None | Glu21Glu | A63G | Hetero | None | None |  | None | None |  |  |
| 23 | 46 | Secundum | None | Glu21Glu | A63G | Hetero | None | None |  | None | None |  |  |
| 24 | 25 | Secundum | None | None | None |  | None | None |  | None | None |  |  |
| 25 | 46 | Secundum | None | Glu21Glu | A63G | Homo | None | None |  | None | None |  |  |
| 26 | 23 | Secundum | None | Glu21Glu | A63G | Hetero | None | None |  | None | None |  |  |
| 27 | 37 | Secundum | None | Glu21Glu | A63G | Homo | None | None |  | None | None |  |  |
| 28 | 51 | Secundum | None | Glu21Glu | A63G | Homo | None | None |  | None | None |  |  |
| 29 | 48 | Secundum | None | Glu21Glu | A63G | Hetero | None | None |  | None | None |  |  |
| 30 | 50 | Secundum | None | None | None |  | None | None |  | None | None |  |  |
| 31 | 45 | Secundum | None | Glu21Glu | A63G | Hetero | None | None |  | None | None |  |  |
| 32 | 36 | Secundum | None | Glu21Glu | A63G | Hetero | None | None |  | None | None |  |  |
| 33 | 58 | Secundum | None | Glu21Glu | A63G | Homo | None | None |  | None | None |  |  |
| 34 | 37 | Secundum | None | Glu21Glu | A63G | Hetero | None | None |  | None | None |  |  |
| 35 | 26 | Secundum | None | Glu21Glu | A63G | Hetero | None | None |  | None | None |  |  |
| 36 | 46 | Secundum | None | Glu21Glu | A63G | Homo | None | None |  | None | None |  |  |
| 37 | 24 | Secundum | None | None | None |  | None | None |  | None | None |  |  |
| 38 | 31 | Secundum | None | Glu21Glu | A63G | Homo | None | None |  | None | None |  |  |
| 39 | 26 | Secundum | None | Glu21Glu | A63G | Homo | None | None |  | None | None |  |  |
| 40 | 19 | Secundum | None | Glu21Glu | A63G | Hetero | None | None |  | None | None |  |  |
| 41 | 20 | Secundum | None | Glu21Glu | A63G | Homo | None | None |  | None | None |  |  |
| 42 | 48 | Secundum | None | Glu21Glu | A63G | Hetero | None | None |  | None | None |  |  |
| 43 | 19 | Secundum | None | Glu21Glu | A63G | Homo | None | None |  | None | None |  |  |
| 44 | 20 | Secundum | None | Glu21Glu | A63G | Homo | None | None |  | None | None |  |  |
| 45 | 27 | Sinus Venosus | None | Glu21Glu | A63G | Homo | None | None |  | None | None |  |  |
| 46 | 48 | Secundum | None | Glu21Glu | A63G | Homo | None | None |  | None | None |  |  |
| 47 | 42 | Secundum | None | None | None |  | None | None |  | None | None |  |  |
| 48 | 41 | Secundum | None | Glu21Glu | A63G | Hetero | Arg138Gln | G413A | Hetero | None | None |  |  |
|  |  |  |  |  |  |  | Gln187His | G561C | Hetero | None | None |  |  |
| 49 | 39 | Secundum | None | Glu21Glu | A63G | Homo | None | None |  | None | None |  |  |
| 50 | 36 | Secundum | None | Glu21Glu | A63G | Homo | None | None |  | None | None |  |  |
| 51 | 43 | Secundum | None | Glu21Glu | A63G | Hetero | None | None |  | None | None |  |  |
| 52 | 24 | Secundum | None | Glu21Glu | A63G | Hetero | None | None |  | None | None |  |  |
| 53 | 36 | Secundum | None | Glu21Glu | A63G | Homo | None | None |  | None | None |  |  |
| 54 | 48 | Secundum | None | Glu21Glu | A63G | Homo | None | None |  | None | None |  |  |
| 55 | 33 | Secundum | None | Glu21Glu | A63G | Hetero | None | None |  | None | None |  |  |
| 56 | 30 | Secundum | None | Glu21Glu | A63G | Hetero | None | None |  | None | None |  |  |
| 57 | 29 | Primum | None | Glu21Glu | A63G | Homo | None | None |  | None | None |  |  |
| 58 | 28 | Secundum | None | Glu21Glu | A63G | Homo | None | None |  | None | None |  |  |
| 59 | 25 | Secundum | None | Glu21Glu | A63G | Homo | None | None |  | None | None |  |  |
| 60 | 18 | Secundum | None | Glu21Glu | A63G | Hetero | None | None |  | None | None |  |  |
| 61 | 22 | Secundum | None | Glu21Glu | A63G | Homo | None | None |  | None | None |  |  |
| 62 | 33 | Secundum | None | Glu21Glu | A63G | Hetero | None | None |  | None | None |  |  |
| 63 | 24 | Secundum | None | Glu21Glu | A63G | Homo | None | None |  | None | None |  |  |
| 64 | 29 | Secundum | None | Glu21Glu | A63G | Hetero | None | None |  | None | None |  |  |
| 65 | 27 | Secundum | None | Glu21Glu | A63G | Hetero | None | None |  | None | None |  |  |
| 66 | 25 | Secundum | SVT | Glu21Glu | A63G | Homo | None | None |  | None | None |  |  |
| 67 | 53 | Secundum | None | Glu21Glu | A63G | Hetero | None | None |  | None | None |  |  |
| 68 | 21 | Secundum | None | Glu21Glu | A63G | Hetero | None | None |  | None | None |  |  |
| 69 | 53 | Secundum | None | Glu21Glu | A63G | Hetero | None | None |  | None | None |  |  |
| 70 | 19 | Secundum | None | Glu21Glu | A63G | Hetero | None | None |  | None | None |  |  |
| 71 | 29 | Secundum | None | Glu21Glu | A63G | Hetero | None | None |  | None | None |  |  |
| 72 | 49 | Sinus Venosus | SND, AVND | Glu21Glu | A63G | Hetero | Arg138Gln | G413A | Hetero | None | None |  |  |
|  |  |  |  |  |  |  | Gln187His | G561C | Hetero | None | None |  |  |
| 73 | 50 | Primum | None | None | None |  | None | None |  | None | None |  |  |
| 74 | 63 | Secundum | None | Glu21Glu | A63G | Hetero | None | None |  | None | None |  |  |
| 75 | 35 | Secundum | None | Glu21Glu | A63G | Hetero | None | None |  | None | None |  |  |
| 76 | 56 | Secundum | None | Glu21Glu | A63G | Homo | None | None |  | None | None |  |  |
| 77 | 63 | Secundum | None | Glu21Glu | A63G | Hetero | None | None |  | None | None |  |  |
| 78 | 33 | Secundum | None | Glu21Glu | A63G | Homo | None | None |  | None | None |  |  |
| 79 | 44 | Secundum | None | Glu21Glu | A63G | Homo | None | None |  | None | None |  |  |
| 80 | 18 | Secundum | None | Glu21Glu | A63G | Homo | None | None |  | None | None |  |  |
| 81 | 50 | Secundum | Junctional rhythm | Glu21Glu | A63G | Homo | None | None |  | None | None |  |  |
| 82 | 21 | Secundum | None | None | None |  | None | None |  | None | None |  |  |
| 83 | 45 | Secundum | None | Glu21Glu | A63G | Hetero | None | None |  | None | None |  |  |
| 84 | 37 | Secundum | None | Glu21Glu | A63G | Homo | None | None |  | None | None |  |  |
| 85 | 31 | Secundum | Atrial fibrilation | Glu21Glu | A63G | Hetero | None | None |  | None | None |  |  |
| 86 | 59 | Primum | Atrial fibrilation | Glu21Glu | A63G | Homo | None | None |  | None | None |  |  |
| 87 | 22 | Secundum | None | Glu21Glu | A63G | Homo | None | None |  | None | None |  |  |
| 88 | 27 | Secundum | None | Glu21Glu | A63G | Homo | None | None |  | None | None |  |  |
| 89 | 23 | Secundum | None | None | None |  | None | None |  | None | None |  |  |
| 90 | 19 | Secundum | None | None | None |  | None | None |  | None | None |  |  |
| 91 | 21 | Sinus Venosus | SVT | None | None |  | None | None |  | None | None |  |  |
| 92 | 26 | Secundum | None | Glu21Glu | A63G | Homo | None | None |  | None | None |  |  |
| 93 | 41 | Secundum | None | Glu21Glu | A63G | Homo | None | None |  | None | None |  |  |
| 94 | 43 | Secundum | SVT | Glu21Glu | A63G | Homo | None | None |  | None | None |  |  |
| 95 | 46 | Secundum | Multifocal AT | Glu21Glu | A63G | Homo | None | None |  | None | None |  |  |
| 96 | 20 | Secundum | None | Glu21Glu | A63G | Homo | None | None |  | None | None |  |  |
| 97 | 12 | Secundum | Atrial tachycardia | None | None |  | Arg138Gln | G413A | Hetero | None | None |  |  |
|  |  |  |  |  |  |  | Gln187His | G561C | Hetero | None | None |  |  |

AA – amino acid change; AT – atrial tachycardia; AVND – atrioventricular node dysfunction; Seq var – sequence variant; SND – sinus node dysfunction; SVT – supraventricular tachycardia.
